# Investigation of the Integration of Strained Ge Channel with Si-Based FinFETs

**DOI:** 10.3390/nano12091403

**Published:** 2022-04-19

**Authors:** Buqing Xu, Guilei Wang, Yong Du, Yuanhao Miao, Yuanyuan Wu, Zhenzhen Kong, Jiale Su, Ben Li, Jiahan Yu, Henry H. Radamson

**Affiliations:** 1 Institute of Microelectronics, Chinese Academy of Sciences, Beijing 100029, China; xubuqing@ime.ac.cn (B.X.); miaoyuanhao@ime.ac.cn (Y.M.); kongzhenzhen@ime.ac.cn (Z.K.); sujiale@ime.ac.cn (J.S.); yujiahan@ime.ac.cn (J.Y.); 2 School of Integrated Circuits, University of Chinese Academy of Sciences, Beijing 100029, China; 3 Beijing Superstring Academy of Memory Technology, Beijing 100176, China; guilei.wang@bjsamt.org.cn; 4 Research and Development Center of Optoelectronic Hybrid IC, Guangdong Greater Bay Area Institute of Integrated Circuit and System, Guangzhou 510535, China; liben@giics.com.cn

**Keywords:** CMOS, FinFETs, Ge selective epitaxy, compressive strain

## Abstract

In this manuscript, the integration of a strained Ge channel with Si-based FinFETs was investigated. The main focus was the preparation of high-aspect-ratio (AR) fin structures, appropriate etching topography and the growth of germanium (Ge) as a channel material with a highly compressive strain. Two etching methods, the wet etching and in situ HCl dry etching methods, were studied to achieve a better etching topography. In addition, the selective epitaxial growth of Ge material was performed on a patterned substrate using reduced pressure chemical vapor deposition. The results show that a V-shaped structure formed at the bottom of the dummy Si-fins using the wet etching method, which is beneficial to the suppression of dislocations. In addition, compressive strain was introduced to the Ge channel after the Ge selective epitaxial growth, which benefits the pMOS transport characteristics. The pattern dependency of the Ge growth over the patterned wafer was measured, and the solutions for uniform epitaxy are discussed.

## 1. Introduction

The miniaturization of devices has been evolving in accordance with the proportional scaling principle proposed by R. Dennard [1]. However, the development of nano-sized devices is hindered by physical limits. Conventional Si-based devices encounter a bottleneck as the pattern keeps shrinking, which has further impact on the reinforcement of the on-state saturation current. To continue the downscaling of technology, previously raised drawbacks, such as parasitic effects, quantum effects, short-channel effects in MOSFETs, the weakening control ability of gate voltage on the carrier concentration of the channel inversion layer and the stringent process requirements, have to be addressed in manufacturing [2,3].

A fin field-effect transistor (FinFET) structure has been proposed to overcome the aforementioned drawbacks. The number of gates in FinFETs can be increased from single to multiple; this helps with the strengthening and coupling between gates, which leads to a better control of carrier transport through the channel. The gate leakage current can be suppressed by SiO_2_ between the gate and the fin [4]. In these transistors, a fully depleted channel and a superior sub-threshold slope performance can be achieved [5,6]. In order to fix the issue of source/drain tunneling, a deep implantation step is sometimes carried out to define the isolation region before the formation of the fin, and the fluctuation of fin height may lead to local isolation failure. Compared with planar devices, FinFETs are also compatible with the complementary metal–oxide–semiconductor (CMOS) process platform, reducing power consumption and improving switching speed without a substantial rise in cost [7,8,9]. Moreover, their vertical design contributes to an integration boost. In this regard, gate-all-around nanowire (VGAANWs or HGAANWs) transistors are designed for 3 nm CMOS nodes [10,11,12,13,14,15,16,17]. However, GeSi/Si multi-layers need to be grown where GeSi is selectively etched [18,19,20] to obtain a 3 nm channel. Monica et al. prepared nanowire-based transistors with state-of-the-art transconductance and electron mobility, and they implemented atomically smooth, mono-crystalline electronic and photonic circuits [21].

**Figure 1:**
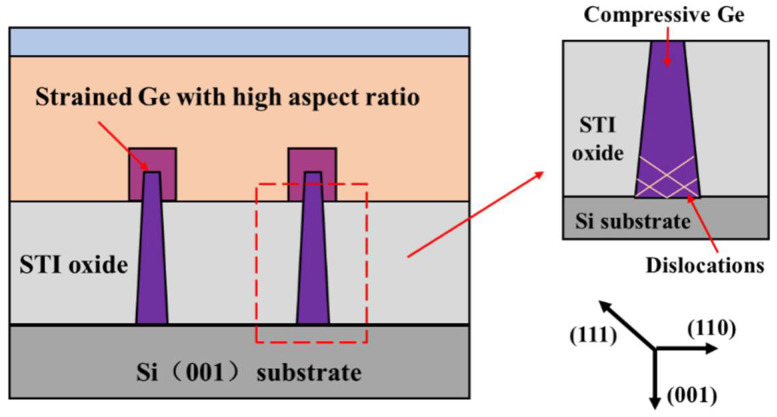
The targeted structure with compressive Ge grown on the high-aspect-ratio trench.

However, equivalent scaling can be realized by increasing carrier mobility for a larger driving current without tuning the gate length of the device. As semiconductor technology is under the projection of ITRS, [22] continues to push forward to nodes below 10 nm, as well as the integration of novel channel materials; e.g., Ge, SiGe and Ⅲ–Ⅴ are under study and promoted in addition to the design complexity of devices. The electron mobility of Ⅲ–Ⅴ compound semiconductor materials is exceedingly high, but their hole mobility is not satisfactory for CMOS circuits [23,24]. In contrast, the hole mobility of Ge, which belongs to group IV elements, is notably higher than that of Si, and there are many similarities between the chemical and physical properties of these two materials, thus making process compatibility extremely convenient. In strained Ge, when Ge atoms match with relaxed substrate materials, they form a compressive strain that is beneficial for hole mobility in general [25,26,27]. The structure of FinFETs is shaped by height, width and sidewall profiles, which is crucial for the strain amount. As a 2D planar channel is transformed into a 3D fin scheme, the critical dimension (CD) of the (110) plane is contracted, but two (110) planes are generated and assist in boosting the channel mobility entirely. If strained Ge is integrated as the channel material, a remarkable gain in the mobility of CMOS devices can be achieved [28]. In recent years, studies have proved that epitaxially growing Ge is one of the most efficient ways to implement strain in sub-14 nm node Ge channel p-FinFETs [29,30,31,32]. Additionally, a numerical analysis of transforming Ge into a material with a direct band was also comprehensively investigated in a discussion on strain in heterostructures [33]. Figure 1 displays a schematic of the designed structures in this study. The sidewall geometry is closely related to anisotropic over-etch when the device is processed. It plays an essential role in the quality of the induction of the strain because the growth of material experiences a more sensitive effect on the sidewall than the center place of the fins [34,35,36,37,38].

During the growth of Ge on Si-fins in the epitaxy module, a two-step growth strategy is adopted. Firstly, a very thin layer is introduced at a low temperature to reduce defects. This buffer layer provides extra nucleation centers for dislocations originating from the big lattice mismatch between Si and Ge. This initial layer effectively assists in decreasing the possibility of 3D island accumulation [39,40,41]. The subsequent growth at high temperatures accelerates the rate of defect motion, which ultimately facilitates annihilation during material growth.

One of the problems in the integration of Ge selective epitaxy is pattern dependency, where the profile of the epi-layer varies over the chip due to layout variation. This problem is also observed on larger scales, e.g., chip to chip or even wafer to wafer. As a result, the linearity of CMOS can be affected [35,36,37,38,42].

This work investigated a novel integration of the high aspect ratio process (HARP) and selective heteroepitaxy of Ge with compressive strain in advanced node FinFETs. Two etching methods were employed to achieve a high-aspect-ratio structure, the wet etching method with tetramethylammonium hydroxide (TMAH) and the in situ dry etching method with HCl. The etching morphologies and rates are discussed. High-quality −2.25% compressive-strained Ge on the fin trench was achieved. Furthermore, the pattern dependency of the Ge deposition over an 8 inch wafer was investigated, and a solution for uniform deposition was presented.

## 2. Experimental Details

**Figure 2:**
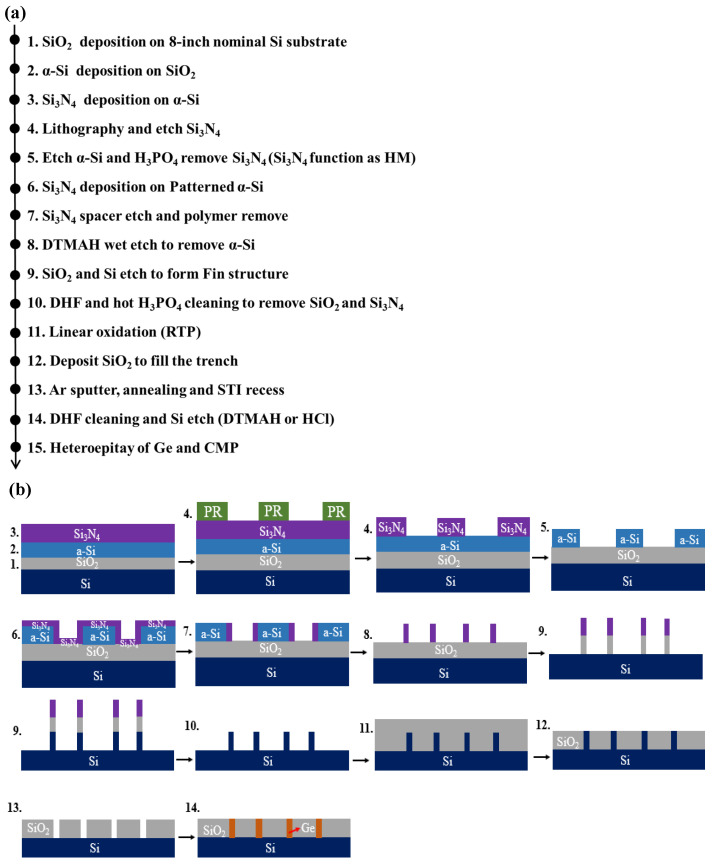
Process flow of the structures used in this research: (**a**) flowchart of the main steps and (**b**) corresponding schematic diagram for (**a**) (not to scale).

All the experiments were performed on p-type Si (001) 200 mm wafers in a reduced pressure chemical vapor deposition (RPCVD) chamber (ASM Epsilon 2000, Almere, The Netherlands). Figure 2 depicts the complete process flow of our experiment. Self-aligned double patterning (SADP) was used to obtain a Si-fin structure with a top width of 20 nm and a height of 110 nm. This process is also known as sidewall transfer lithography (STL) [43,44,45]. Silicon oxide, amorphous silicon (α-Si) and silicon nitride were deposited on the nominal Si substrate in sequence (steps 1–3) using thermal furnace method and plasma-enhanced CVD (PECVD) reactor (D250L, Corial, France), and then the pattern was transferred to the α-Si using photolithography and etching (steps 4–5). The amorphous Si mandrel formed at this stage determined the pitch of the fin. A silicon nitride film with good conformal coverage was deposited on the patterned samples (step 6), and then spacer etching was carried out (step 7). The mandrel made of α-Si was wet etched with TMAH solution, leaving the silicon nitride spacer on both sides of the α-Si to form dummy fins on the surface of the substrate so as to double the density of the patterns. Silicon nitride/silicon oxide spacers of 20 nm width were applied as a hard mask (HM), which was used to etch the silicon below, and fin shapes with different aspect ratios determined by the etch depth and sidewall width were prepared (steps 8–9). Residual silicon nitride and silicon oxide in the head of the dummy fins were removed with hot phosphoric acid (H_3_PO_4_) and diluted hydrofluoric acid (DHF), respectively (step 10). Next, a thin oxide layer was deposited on the surface of the Si-fin using rapid thermal processing (RTP), aiming to repair the damage caused by the plasma etching to the dummy fin and to improve the roughness of the surface. This was followed by the shallow trench isolation (STI) module, where 200 nm silicon oxide film was grown (step 12), and chemical mechanical polishing (CMP) was utilized to planarize the topography. Through the combination of dry etching and wet etching with DHF (1/100 HF), the silicon oxide was thinned and recessed, which enabled the top of the fin to be exposed for the ensuing dummy fin etching study (steps 13–14). In the whole process, only one step of optical exposure was taken, supplemented by etching, film deposition and other processes to realize the transfer of patterns, greatly easing the stringent requirements of the equipment.

Taking advantage of the difference in the etch selectivity to silicon oxide and the head of Si exposed on its plane, 25% TMAH solution diluted in 1:10 deionized water at room temperature and in situ HCl dry etching under a high temperature before epitaxy were used separately. In the DTMAH solution with the same concentration, we recorded the etching morphology and rate under corresponding etching times of the previously processed high-aspect-ratio structure. Similarly, the temperature remained stable at 850 ℃, while the input gas flow of HCl (used as etching gas) was kept constant at 2 slm (standard liters per minute). The growth of Ge on the high-aspect-ratio trenches was performed using a two-step method. All samples experienced a standard cleaning procedure prior to epitaxy (SPM followed by APM with DHF at the end) [46]. The load locks were pumped down as soon as they were transferred into the equipment in order to avoid any potential contamination on the wafer surface.

The outstanding feature of the scheme is that the threading dislocations perpendicular to the channel generated in the growth can be guided to the STI sidewall with the help of the facet epitaxially formed in the high-aspect-ratio trench, thereby blocking these defects at its bottom and ensuring that the quality of the channel area is improved. Relying on this aspect ratio trapping (ART) technology, the steps of eliminating epitaxial material defects through additional thermal annealing after conventional epitaxy could be skipped, which offers a larger window for reducing the thermal budget of the whole integration process.

The cross-section of the processed Si-fins was analyzed using a high-resolution scanning electron microscope (HRSEM) to determine any possible damage to the shape of the Si-fins during the manufacturing steps. A transmission electron microscope (TEM) was utilized to investigate the eventual defect situation and lattice distortion in the Ge epi-layers. High-resolution X-ray diffraction (HRXRD) was used to investigate the strain and crystal quality, and energy-dispersive spectroscopy (EDS) was also employed to determine the Ge profile in these samples.

## 3. Results and Discussion

### 3.1. High-Aspect-Ratio Fin Structure Formation

With the shrinkage of device dimensions, a narrowed fin width ameliorates the short-channel effect to realize a better electrostatic characteristic, but it is also influenced by the quantum effect and the deterioration of the surface roughness in the channel, leading to a decrease in mobility and the driving current. Moreover, the micro-trench undergoing STI recess will have its height shortened. Additionally, HF wet cleaning to remove native oxide before the selective epitaxy is isotropic results in the loss of sidewall and expands the fin width. One compensation for the above issues is to raise the height of the fin to create a higher, narrower and steeper shape, which broadens the process tolerance and optimizes the performance in the effective current and the effective gate capacitance. When preparing FinFET structures under this STI-first scheme, a dummy Si-fin with a high aspect ratio is needed, the morphology of which has a direct impact on the following etching and epitaxy quality of Ge-fins.

**Figure 3:**
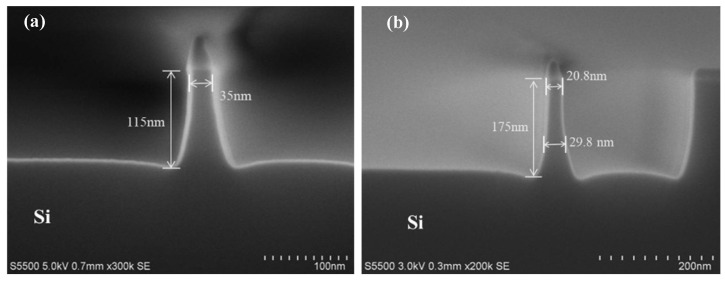

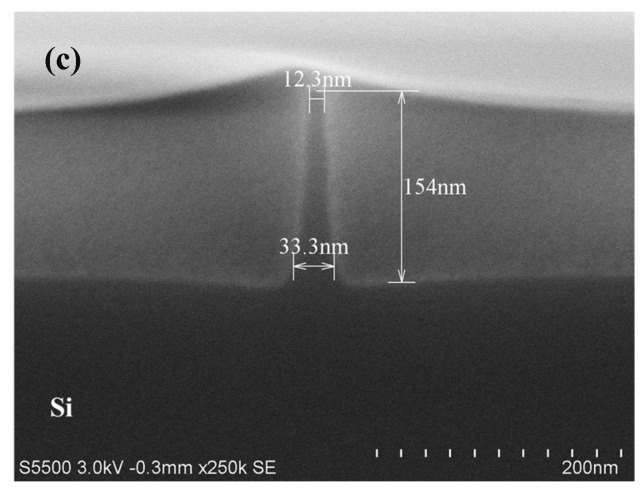
Si-fins with different aspect ratios: (**a**) height ≈ 115 nm, width ≈ 38 nm; AR ≈ 3:1; (**b**) height ≈ 175 nm, width ≈ 23 nm, AR ≈ 8:1. (**c**) The structure in (**b**) after the removal of HM, STI filling and dry etching (height ≈ 154 nm, width ≈ 20 nm, AR ≈ 8:1).

Figure 3 illustrates the Si-fin profile after etching the silicon nitride spacer used as a patterning hard mask (HM). During the etching process, CF_4_ was used to open the native oxide. The oxygen was ionized into oxygen plasma at a pressure of 10 mTorr after ignition, and then the main etching step was performed. By optimizing parameters such as the etching gas flow, gas composition, etching time and bottom electrode bias, Si-fins with aspect ratios of 3:1 (height ≈ 115 nm and width ≈ 38 nm) and 8:1 (height ≈ 175 nm and width ≈ 23 nm) were obtained, as depicted in Figure 3a,b, respectively. As shown in Figure 3, Si-fins with a high aspect ratio, a flat and steep topography and a restrained footing figuration at the bottom were prepared. The significantly increased effective gate area is mainly contributed to by fin height, and, therefore, fin pitch and the contacted gate pitch in each standard cell unit occupied an extended miniaturization space for transistors.

### 3.2. Dummy Fin Removal: Wet Etching and In Situ Dry Etching

Wet cleaning with H_3_PO_4_ and DHF was carried out successively to remove the silicon oxide and the silicon used as a HM on the top of the Si-fins, as well as the residual polymers produced by dry etching. Next, RTP was performed to realize the thermal treatment, which not only repairs the damage to the fin that originated from the previous etching but also makes the Si head round to avoid point discharge. Another point is that rapid thermal oxidation could form a thin capping layer of silicon oxide (~30 Å) on the Si surface, which serves as the seed layer for STI filling, offering better conformal coverage. STI filling in HARP was implemented by employing sub-atmospheric chemical vapor deposition, using the reaction of tetraethyl orthosilicate (TEOS) and O_3_ in a large flow to deposit silicon oxide with a thickness of 3000 Å. The STI oxide was thinned by employing Ar^+^ sputtering, where the argon gas was ionized with glow discharging, and then bias voltage was applied to accelerate these ions to planarize the surface. Figure 3c shows the topography after the removal of HM, linear oxidation, STI deposition and Ar^+^ dry etching of the structure shown in Figure 3b. RTP, in addition to measurement variations, may lead to slight deviations in the height and width, which does not affect the discussion. After polishing, annealing at 1050 ℃ for 30 min in N_2_ atmosphere was supplemented to alleviate the lattice damage in the shallow surface and to lower the risk of leakage caused by the dissociation of the oxide layer. However, the controllability of DHF on the corrosion rate to STI can be enhanced after densification, and insulation failure due to the excessive loss of dielectric is also avoided. Annealing at a high temperature in this step prevents harm to followed up Ge-fins and the negative effect of Ge on densification, which makes such integration methods more desirable. In our experiments, the annealed samples were rinsed for 45 s with HF diluted at a ratio of 1:100 (DHF) to expose the Si head of the dummy fin for further etching processes. It is necessary to note that the rinsing time should be carefully chosen to restrain the undue consumption of STI; otherwise, it may cause the loss of the aspect ratio. 

**Figure 4:**
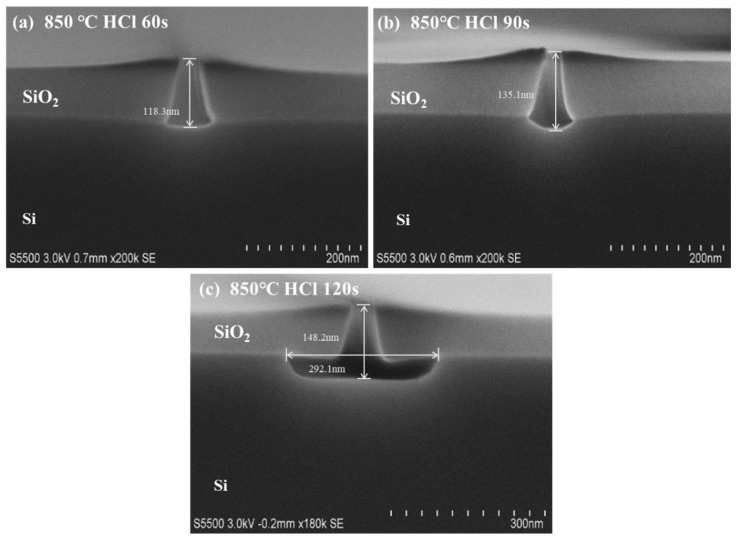
Depth and morphology of in situ HCl dry etching under different experimental times at 850 ℃: (**a**) 60 s; (**b**) 90 s; (**c**) 120 s.

Figure 4a exhibits a cross-sectional SEM view of the dummy fin. It can be seen that an ideal Si-fin has a height of 118 nm and a width of 37 nm. The top of the fin was exposed through the modified DHF rinsing. Both the in situ HCl dry etching before the growth of channel material in RPCVD and DTMAH wet etching are capable of corroding Si-fins in STI. The heteroepitaxy of Ge is directly coupled to the etched substrate, and the growth quality is intensely related to the recess profiles.

After the sample was transferred into the reactor, the temperature was kept at 850 ℃, while the HCl partial pressure was kept constant at 2 mtorr. The dry-etched Si-fin morphologies with different etching times are displayed in Figure 4. It was found that the designed Si-fin with a height of 110 nm (AR = 3:1) was completely removed after 60 s, and the topography showed a trapezoidal shape, which is the same as that of the dummy fin at the start due to the superior selectivity of HCl dry etching. When the time was prolonged to 90 s, the Si-fin in the trench was completely etched to the foot (etch depth ≈ 135 nm); however, the HCl continued to act, rounding the bottom as shown in Figure 4b. If the duration is further increased, taking 120 s as an example, an undercut could occur for Si in all directions, which is unacceptable because it is detrimental to the epitaxy, and the isolation will be spoiled (etch depth ≈ 148 nm and etch length ≈ 292 nm in the (110) direction). The etching rate and the generation of defects are highly sensitive to the structure in dry etching, and the loading effect may introduce multiple facets with diversified crystal planes at the bottom of the trench. A minor change in the shape will trigger obvious fluctuations in the etching performance, especially for those with small sizes and a high aspect ratio.

**Figure 5:**
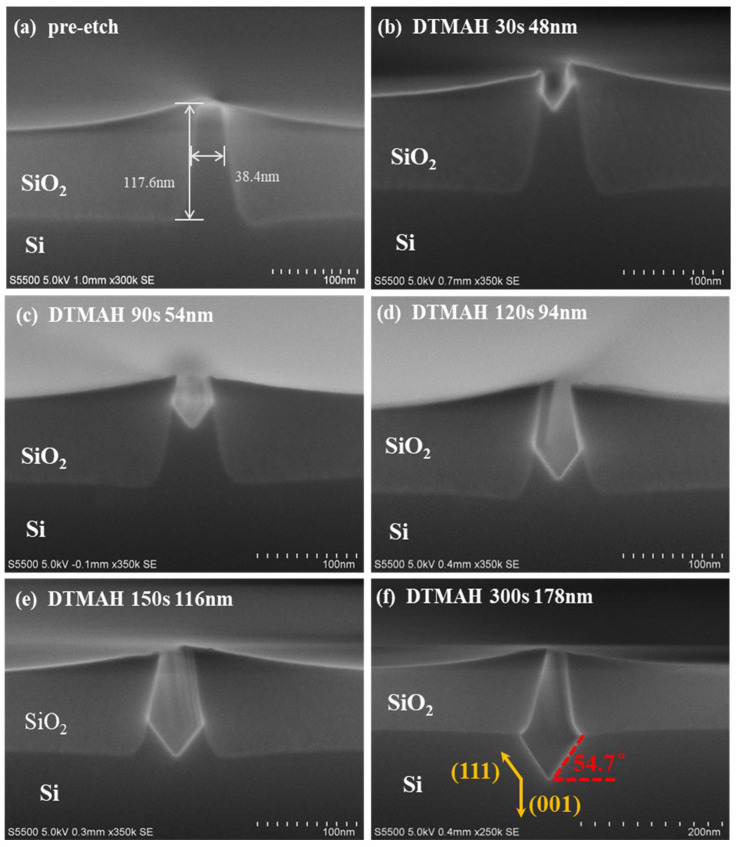
Depth and morphology of 2.5% DTMAH wet etching under different experimental times at room temperature: (**a**) before etching; (**b**) 30 s; (**c**) 90 s; (**d**) 120 s; (**e**) 150 s; and (**f**) 300 s.

Figure 5 displays the evolution of the morphology of the samples etched using DTMAH at different times. Specimens with the same structures as those used in previous experiments were immersed in 2.5% DTMAH solution at room temperature, and the chemical reaction of Si in DTMAH can be described as Equations (1) and (2).

(1) $$\mathrm{Si}+2{\mathrm{OH}}^{-}\to \mathrm{Si}{\left(\mathrm{OH}\right)}_{2}^{2+}+4e^{-}$$

(2) $$\mathrm{Si}{\left(\mathrm{OH}\right)}_{2}^{2+}+4e^{-}+4H_{2}O\to \mathrm{Si}{\left(\mathrm{OH}\right)}_{6}^{2-}+2H_{2}$$

Figure 5a presents the pre-etched Si-fin. It is clearly shown that a V shape is formed as the etching time gradually increases from 30 s to 300 s in Figure 5a–f. This is due to the difference in the etching rate of TMAH anisotropic corrosion on Si (001) and (111) directions. In the beginning, the Si (001) direction dominates the etching, and the corrosion extends along <001>, which is perpendicular to the substrate. The V-shaped geometry is initiated within 30 s until the sidewall boundary of STI is eroded, and then the (111) plane is turned to guide the etching as the dummy fin is consumed. Compared with the flat-bottomed structure engendered by HCl, the V shape formed by DTMAH is beneficial to the suppression of dislocations due to the lattice mismatch in epitaxy. The etching rates of these two methods with different etching times are listed in Table 1 as a reference.

Although the heterogeneous preparation of Ge or SiGe on Si will produce more defects on the (111) plane than on (001), these uniformly distributed defects are parallel to (111) of the trench, indicating that they would eventually be captured by the sidewalls in STI instead of propagating to the top of the material, which paves the way for denser integration with smaller devices. Additionally, fewer defects appear on (111) than on (001) for Ⅲ–Ⅴ group materials, and the V shape boosts the growth quality for both group IV and group Ⅲ–Ⅴ materials, which, in turn, holds the potential to promote the integration of n-type and p-type devices with different channel bases.

### 3.3. Ge Selective Epitaxy

**Figure 6:**
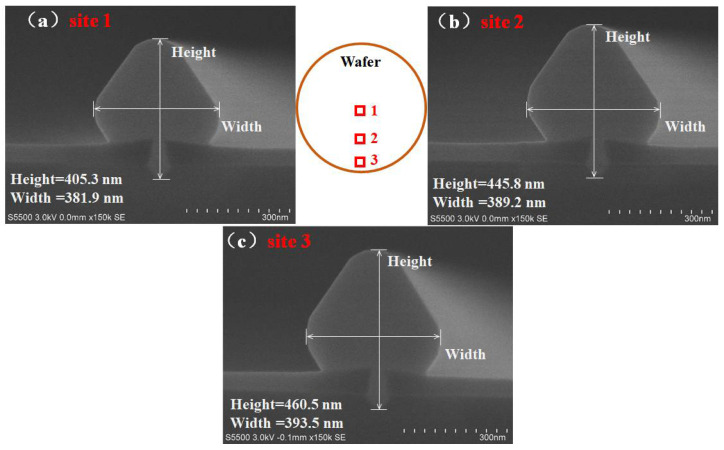
Cross-section of selectively grown Ge selected in different parts of the wafer: (**a**) site 1, the center; (**b**) site 2, between the center and edge; (**c**) site 3, the edge.

Selective epitaxy is an attractive deposition technique for device application, and it is especially aimed at source/drain ultra-shallow junctions [47,48,49]. The deposition of Ge on Si-fins is not only a very critical step, but it is also a sensitive step in STI-first technology. The layer quality may be degraded by any undesired residual species (after the etching step) or native oxide. Thus, ex and in situ cleaning are important steps prior to epitaxy. The aspect ratio and the overall size of STI structures have gone a step further, and the issue to be overcome in Ge filling is the aggravation that goes with it. Pattern dependency was a major concern as we conducted the selective epitaxy of Ge [50,51]. Kinetic models and experimental explorations have been reported in various publications, but an effective method that is capable of completely eliminating pattern dependency has not been demonstrated to date [52,53,54,55]. As shown in Figure 6a–c, three sites were selected from the center to the edge of the wafer after Ge growth. It can be seen in Figure 6 that the height and width of site 1, which is located in the center, are slightly narrowed. The size of the Ge mushroom increased gradually from the center to the edge, which signifies that the growth rate also had a modest rise due to gas kinetics and non-linear gas consumption over the patterned wafer. The dimensions of the two outer parts were close, and the overall uniformity was good. In the following discussion, sample quality is characterized and analyzed.

**Figure 7:**
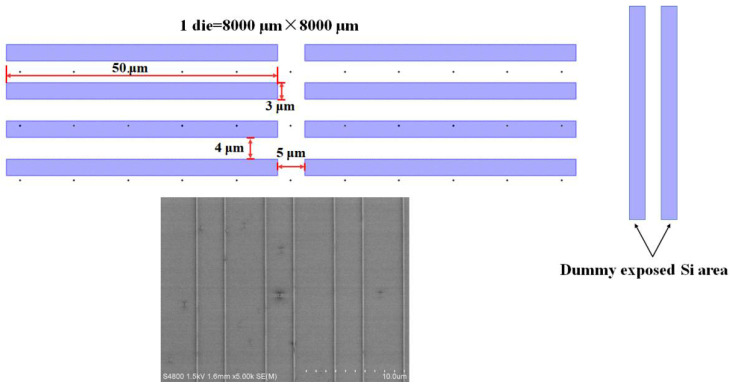
Layout chip of the fins and plan view of the fins for selective epitaxy. The exposed Si area to the total chip area is almost 1%. The exposed area of the chip has to be compensated by dummy openings at the edge of the wafer.

The main reason behind the pattern dependency is the gas consumption over the chip. Therefore, the exposed Si area to total chip area (or Si coverage) has a crucial role in gas consumption and has to be kept constant for a uniform deposition. Figure 7 shows the chip layout of a die over the 8 inch wafer. The Si coverage was only 1% in our experiments. This means that kinetic growth is very vulnerable to Si coverage, which becomes smaller at the edge of the wafer. In order to keep a uniform epitaxy profile, the Si coverage has to be compensated by 1% using dummy strips preferably at the edge of the die as shown in Figure 7. The area and the number of strips depend on the actual extra oxide area.

Another key point is that the introduction of a high amount of HCl assists in decreasing the pattern dependency in selective epitaxy. This idea is used to furnish a uniform deposition over the whole wafer by lowering the lateral flow over the oxide surface toward the fin regions [56,57]. Unfortunately, such a solution leads to a low growth rate.

**Figure 8:**
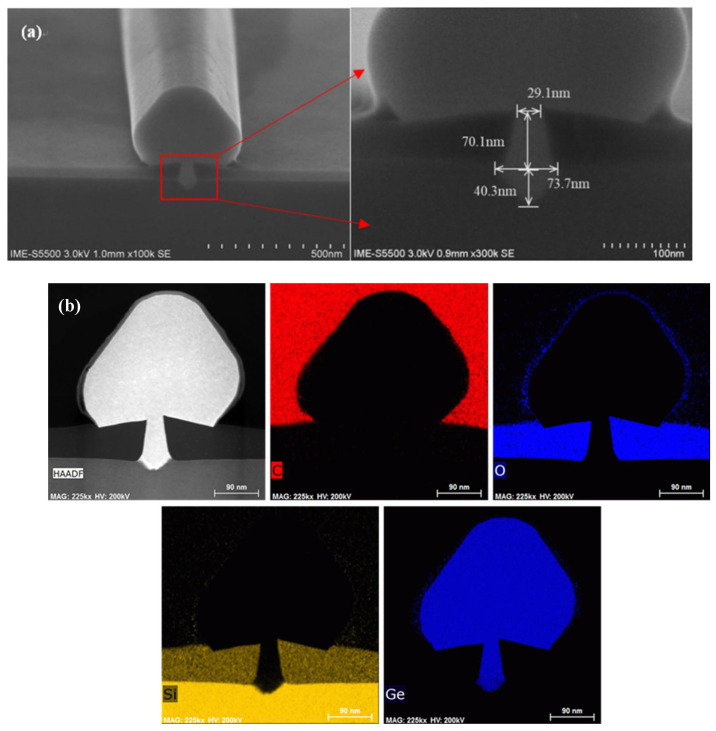
(**a**) Cross-sectional SEM image of the Ge-fin structure; (**b**) EDS mappings of the Ge filling in STI with elements of C, O, Si and Ge in order.

A two-step procedure was carried out to prepare the Ge channel. First, a low-temperature nucleation layer (buffer layer) was grown at 400 ℃ under 100 Torr for 60 s to alleviate the dislocations introduced by lattice mismatch. Benefiting from the distinctive advantage of the V shape for defects filtering on the (111) facet by DTMAH recessing, dislocations were confined to the interfaces of the bottom area. Then, the temperature was increased to 650 ℃ under the same pressure for the 60 s Ge deposition. As soon as the growth finished, the samples were baked at 850 ℃ for 180 s in the chamber. HCl was imported during the whole epitaxy to ensure high growth selectivity for the polycrystalline Ge on the silicon oxide as shown in Figure 8a and to avoid film accumulation to maintain a clean quartz chamber. Figure 8b shows the EDS analysis at the region where the growth of Ge is completed. The results indicate that the boundary of each structure reached an agreement with the design, and there was no evident diffusion of elements. Thus, this is an effective way to integrate a high aspect ratio of Ge channel material into advanced FinFETs using DTMAH etching followed by the Ge selective epitaxy process.

**Figure 9:**
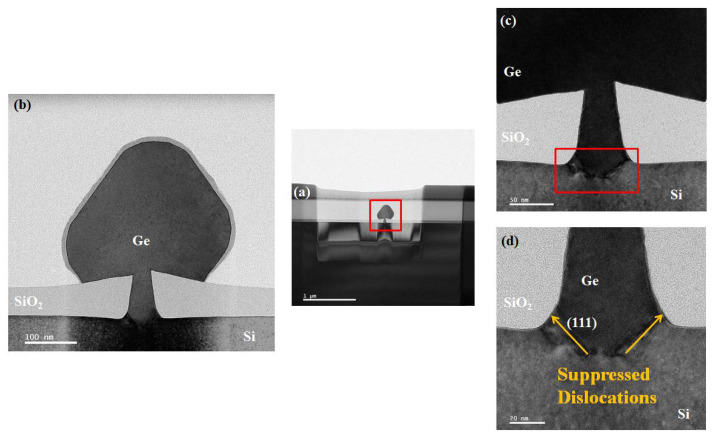
(**a**,**b**) Cross-sectional TEM image in the bright field of the selectively grown Ge-fin structure; (**c**) from the bottom of (**b**): interface between selectively grown Ge layers at the sides and bottom of silicon oxide mask; (**d**) magnified image of (**c**): clear illustration of the dislocation being suppressed by silicon oxide.

ART works on the processed high aspect ratio of STI to annihilate the threading dislocations caused by the mismatch in heterojunctions, such as Ge/Si in our case, at the lower part of the fin, thereby guaranteeing that the head of the fin as the carrier channel will not be dragged by the defects, as illustrated in Figure 9. More specifically, the growth rate of heteroepitaxial material varies in different directions, so the crystal facet inclined to the substrate in relative could be formed in the period of the growth (Ge ($1\bar{1}1$) and ($1\bar{1}3$) as an example). As Ge precipitates, the vertical plane in the trench is depleted by the facet, and the stretching direction of the threading dislocation at the interface is a function of the Burgers vector and the material growth direction, which is almost perpendicular to the growth crystal plane for the most part. Zoomed in images of the red frame in Figure 9a are presented in Figure 9b–d. In Figure 9c,d, it is vividly shown that the defects spread along the (111) direction around the interface between the Si substrate, and Ge is blocked when encountered with the silicon oxide standing on both sides of the boundary. Additionally, no other severe defects were found after the inspection by TEM, revealing a high-quality Ge selective heteroepitaxy based on Si.

**Figure 10:**
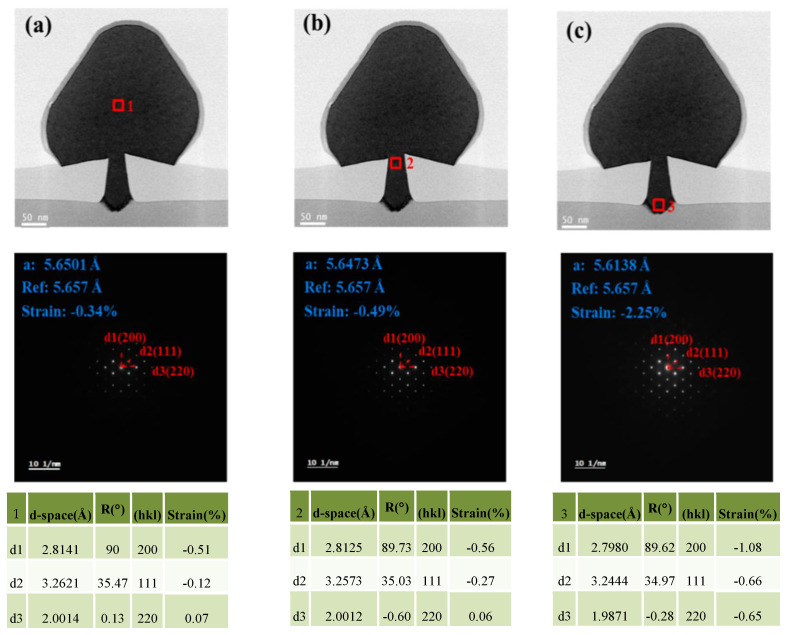
Cross−section HRTEM images of the Ge epi-layer: (**a**) above the fin head, (**b**) located in the middle (around the fin top) and (**c**) bottom part of the channel. The electron diffraction patterns of different regions are presented in each group.

To unveil the strain state in the heterogeneously prepared Ge channel, the electron diffraction pattern of TEM was conducted. Figure 10 depicts the selected area electron diffraction (SAED) patterns of sites 1, 2 and 3 marked in red. Formula (3) was used to calculate the lattice spacing *d* in different parts of the Ge channel as follows:

*Rd = Lλ* (3)

where *R* is the length of the camera, *λ* is the wavelength of the incident light, and *L* is the spacing of the crystal planes in accordance with the diffraction bands. The *d*-values of the three sites are shown in the tables. Afterward, the lattice constant *a* can be easily inferred on the basis of the different lattice spacings, *d*1, *d*2 and *d*3. The lattice constant *a* of sites 1, 2 and 3 are 5.6501 Å, 5.6473 Å and 5.6138 Å, respectively. Compared with the lattice constant of the standard bulk Ge, namely, 5.6578 Å, the strains over the fin top, in the middle part and in the bottom part as shown in the selectively grown Ge are −0.34%, −0.49% and −2.25%, respectively. This outcome verifies that the upper part of Ge possesses a compressive strain. The compressive strain gradually releases with the increase in Ge thickness along the (001) direction through the whole trench from the bottom to the top, and the prominent strain is observed at the foot.

The strain originating from the lattice mismatch is considered to achieve full relaxation by misfit dislocations that nucleate at the free surface. Generally speaking, the critical thickness (about 1 nm) of Ge grown on Si and the huge difference in the thermal expansion coefficient between Ge and Si together with STI oxide in the lateral direction constitute a source of the strain. The thermal coefficient of Ge (5.8 × 10^−6^ K^−1^) is much higher than those of SiO_2_ (0.5 × 10^−6^ K^−1^) and Si (2.6 × 10^−6^ K^−1^); the volume change of Ge is subsequently more than that of the other components in the structure when cooled from the post-growth baking temperature of 1123 K to 300 K, especially relative to STI oxide, which accounts for the introduction of strain. ART technology facilitates the limitations of the threading dislocation movement on the glide planes of (111) and (113) by the sidewall in our research, and, thus, the value of compressive strain decreased along the parallel direction to the grooves.

**Figure 11:**
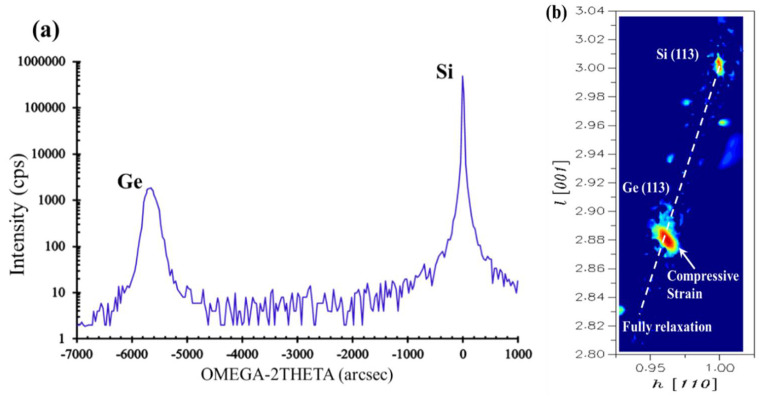
(**a**) HRXRD rocking curves (RCs) around (004) reflection and (**b**) HRRLMs around (113) reflection for the selectively grown Ge.

Figure 11a depicts the HRXRD result around (004) of the Ge channel integrated directly on Si, and the full width at half maximum (FWHM) of Ge peaks is about 187 arcsec. In order to further check the strain in this sample, a high-resolution reciprocal lattice map (HRRLM) around the (113) reflection was collected, as shown in Figure 11b. It is revealed that Ge grown on the trench with the V-shaped bottom contains compressive strain for the peaks located on the right side of the fully relaxed boundary. The material characteristics reflected by these X-ray characterizations are consistent with the conclusions deduced from TEM. On the basis that such a core structure and modified process scheme were developed, it is believed that reliable convenience and solid possibility are provided for the integration of the novel device with impressive and powerful characteristics.

For the proposed process in this work, the scalability is not difficult; however, the variability over the wafer warrants further investigation. Both the selective epitaxy and etching suffer from pattern dependency, and any minor change in the chip layout affects the profile. Since the main reason for pattern dependency is the non-uniform consumption of the precursor molecules over the chip, the exposed Si area has to be kept constant. This is especially the issue at the edge of the wafer, where there is less exposed Si area compared to the center of the wafer. We need to compensate the exposed Si area in the chip layout in order to control pattern dependency. Therefore, we believe that our process can be an appropriate candidate for the CMOS process in the near future.

## 4. Conclusions

We proposed the integration of a strained Ge channel with Si-based FinFET fabrication in which the formation of high-aspect-ratio dummy fins, two etching methods and selective Ge heteroepitaxy are involved. The samples were characterized by SEM, TEM, EDS, HRXRD and HRRLMs to analyze the structure topography processed by various etching conditions, defect propagations and strain distributions of grown Ge. The corresponding corrosion rates and features were summarized. After a series of optimized processes emphasized in the discussions, we reaped dummy Si-fins with a delicate shape with aspect ratios of 3:1 and 8:1. Combined with the 300 s of DTMAH wet etching, high-quality Ge with compressive strain was prepared on V-shaped grooves with a two-step growth method. Furthermore, the Ge growth rate increased for the chips closer to the edge of the wafer. This non-uniformity growth rate is a result of pattern dependency, which can be compensated when exposed dummy Si areas are inserted in the chip closer to the edge. This research establishes meaningful developments and conclusions for the integration of selective epitaxy of pure Ge on the channel of FinFETs.

## Figures and Tables

**Table 1:** Etching rates of the two methods at different times.

Etch Method	Etch Time (s)	Etch Depth (nm)	Etch Rate (Å/s)
DTMAH wet etching	30	48	16
	90	54	6
	120	94	7.83
	150	116	7.73
	300	178	5.93
In situ HCl dry etching	60	118	19.7
	90	135	15.0
	120	148	12.6

## Data Availability

The data is available on reasonable request from the corresponding author.
